# Strength Training and Detraining in Different Populations: Case Studies

**DOI:** 10.2478/v10078-011-0052-7

**Published:** 2011-10-04

**Authors:** Mário C. Marques, Adam Zajac, Ana Pereira, Aldo M. Costa

**Affiliations:** 1 Department of Sport Sciences, University of Beira Interior (UBI), Covilhã, Portugal; 2 Department of Sport Sciences, Exercise and Health, University of Trás-os-Montes and Alto Douro (UTAD), Vila Real, Portugal; 3 Research Centre for Sport Sciences, Health and Human Development (CIDESD), Vila Real, Portugal; 4 Department of Sports Training, Academy of Physical Education, Katowice, Poland

**Keywords:** strength, power, detraining, athletes, physical students, youth

## Abstract

Many researchers have demonstrated that a specific strength training program can improve maximal strength and, the rate of force production, reduce the incidence of muscle-skeletal injury, and contribute to faster injury recovery times, thereby minimizing the number of missed practice sessions or competitions. Yet, to our best knowledge, there is no apparent consensus on the appropriate method of muscle strength and power training to enhance performance in distinct populations groups. Interruptions in training process because of illness, injury, holidays, post-season break or other factors are normal situations in any kind of sport. However, the detraining period and its consequences are not well reported in sports literature, and namely during puberty. Therefore, the aim of this paper was to discuss several case studies concerning different populations such us physical students, age-swimming competitors and elite power athletes.

## Introduction

During the last 50 years, muscle strength training (ST) has been a major topic for coaches, athletes and researchers (Marques and González-Badillo, 2006). However, despite increasing professionalization, there is a paucity of research data concerning performance in elite athletes. Two main reasons for this may be suggested. The first reason is due to the fact that experimental studies on elite sportsmen are very difficult to carry out for many reasons. The second reason is ethical, being related to the inclusion of a control group in the study design of elite athletes. This is because the withholding of potentially important training would be detrimental for the development of the players so selected. Such considerations ought not to detract from the necessity and importance of this type of research in strength and power events (Marques et al., 2008). Unfortunately, there is no apparent consensus on the appropriate method of strength and power training to enhance performance, especially in power sports.

Beyond high-level sport, scientific evidence also indicates that ST should be part of a comprehensive health maintenance and specific performance, as long as it is carefully prescribed and monitored (Faigenbaum et al., 2009). Moreover, a positive relationship between physical activity and aerobic fitness performance has been also established in adults; whereas in children, this relationship seems less clear (Malina, 2001). Nevertheless, it was reported by several studies that physical activity levels of children aged 10 to 15 years old are positively associated with physical fitness (Watts et al., 2004). Fortunately, there is strong evidence that school-based interventions are effective in promoting physical fitness levels, and therefore school seems to provide an excellent place to enhance it by implementing ST programs. Furthermore, muscle strength and endurance training are often performed concurrently in most exercise programs in wellness, fitness and rehabilitative settings, in an attempt to reach different physical fitness goals (Shaw et al., 2009). Several studies have shown that simultaneously performing resistance and cardiovascular training may impair the strength gains achieved by ST alone (Shaw et al., 2009; Garrido et al., 2010). However, to the best of our knowledge, very few studies have examined the effects of resistance training with concurrent resistance and endurance training on muscular strength development.

This scarcity of literature is hardly comprehensible because combined intervention of strength and aerobic training is a common practice in many sports and on daily routines. For example, overall performance depends heavily upon muscular strength and aerobic enhancement, especially at competition level (Leveritt et al., 2000). Even so, some studies showed that concurrent strength and endurance training regimens seem to inhibit strength and power development when compared with strength training alone (Dudley and Djamil, 1985; Abernethy and Quigley, 1993; Hennessey and Watson, 1994), but scientific literature has produced inconclusive data. On this, competitive swimming is an example of such combination, but the scientific evidence is still scarce (Aspenes et al., 2009, Garrido et al., 2010).

Another topic that still needs further clarification is associated with the discontinuation of training sessions because of illness, injury, vacancies, post-season break or other factors normal in any kind of sport. A reduction of physical activity level is often reported (Hortobagyi et al., 1983; Marques and Gonzalez-Badillo et al., 2006). Yet, the detraining period (DT) and its consequences are not well reported in sports literature, in either youth or in elite sports populations. Furthermore, a period of overload decrement (strength training cessation) could produce a positive delay transformation to enhanced specific performance (Zatsiorsky, 1995).

In view of the foregoing, the main purpose of this paper was threefold: (i) to review the effects of strength training programs in distinct populations; (ii) to review the effects of combined strength and aerobic training for increasing upper and lower body strength, power and performance in youth; (ii) and to review the effects of distinct detraining periods (strength training cessation) on strength and specific performance amongst different groups of individuals.

## Strength Development:

### Elite Power Team Sports: a case study

From the various training variables, it appears that training intensity is the most important to be consider when designing a ST program to target maximum dynamic strength in high level athletes (Marques et al., 2006). On this issue, research has shown that RT with external loads corresponding to 80–100% of one repetition maximum (1RM) is most effective for increasing maximal dynamic strength because this loading range appears to maximally recruit muscle fibers and produce further neural adaptations (González-Badillo et al., 2005). Between this intensity range of 80–100% of 1RM, experienced weight-trained athletes routinely invest their RT time in the use of excessively heavy loads (i.e., > 90% of 1RM) (Marques et al., 2006), because it is commonly believed that effective increases in maximal strength can be achieved by training at these relative intensities. Marques and González-Badillo (2006) reported that a short-term ST (e.g., 12 consecutive weeks) using moderate relative intensity tended to produce significant enhancements in top team handball players’ performance in parallel squat and concentric bench press. These conclusions should, however, be interpreted within the context of this population of experienced athletes. Nevertheless, it is not known whether optimal intensity stimulus at these extremely heavy loads is effective for the development of maximal dynamic strength in elite power sportsmen. To date, the optimal volume stimuli for the development of strength and the effectiveness of stimuli within the training process have not been satisfactorily ascertained by the scientific community (González-Badillo et al., 2006). In fact, several studies indicated that one set per exercise or three sets can be equally efficient in strength enhancement whereas others have reported that only RT with multiple sets contributed to obtain better results (Rhea et al., 2003). These results could possibly contribute to the variable outcomes of previous studies with respect to the RT experience of participants. Therefore, for example, non-elite athletes can respond favorably to one or more sets per exercise, especially during the initial training weeks. In contrast, experienced RT participants can only increase strength values by performing higher training repetitions. Marques et al. (2006) showed that professional volleyball players can increase maximal dynamic strength performance (1RM) using low volume and medium/high intensity. After 12 consecutive weeks of RT, an increase of 1RM bench press (1RMBP) and 4RM squat (4RMPS) was observed, corresponding to 15 and 19% (p<0.01), respectively. Consequently, all the athletes were in good overall condition. This strategy requires that each repetition be performed at relatively high speed, on the premise that greater gains in muscular power will be achieved with each repetition. Thus, increasing training volume does not always provide a better stimulus for improving adaptations during a long-term competitive period. These observations may have important practical relevance for the optimal design of ST programs for trained athletes. During the experimental period (Table 1), average training efficiency in 1RMBP and 4RMPS was 0.07 %·lift^−1^ and 0.1 %·lift^−1^ respectively. No differences were observed in training efficiency between the first half (1–6 weeks) and the second half (7–12 weeks) of the training period. Training Efficiency. To quantify the effort to benefit ratio, training efficiency was defined as the average percentage gain in bench press and squat performances during the 12-week training period divided by the total number of repetitions lifted at loads greater than 80% of 1RMBP and 4RMPS, respectively.

### Competitive Age-Swimming: a case study

**Figure 1:**
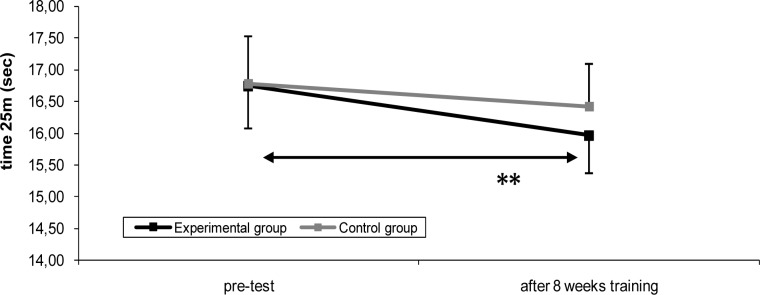
Swimming performance in 25 m front crawl at the beginning of the protocol (pré), after eight weeks of training (post) for the experimental and the control group. Solid lines and * represent differences between evaluation moments in the experimental group. Solid lines represent differences between evaluation moments in the experimental group * p<0.05. (after Garrido et al., 2010).

Several studies showed that concurrent training compromises the development of strength and power but does not effect the development of aerobic conditioning when compared with either form of stand-alone training (Dudley and Djamil, 1985; Hennessey and Watson, 1994). However, other researchers have reported that concurrent training has an inhibitory effect on the development of strength and endurance (Sale et al., 1990; Abernethy and Quigley, 1993). More recently, Aspenes et al. (2009) and Garrido et al. (2010) examined the effect of a training (twice a week) intervention consisting of a concurrent strength and endurance training among age-swimmers. Aspenes et al. (2009) observed that the intervention group improved in dry land strength, tethered swimming force and 400 m freestyle performance more than the control group. No changes were observed in technical variables (i.e., stroke length, stroke rate) and performance in short-distance events (50 and 100 m freestyle). Regarding the young swimmers, Garrido et al. (2010) showed that a combined strength and aerobic swimming training allow dry land strength developments in young swimmers. The main data cannot clearly state that strength training allowed an enhancement in swimming performance (Figure 1), although an improve trend for the sprint performance due to strength training was noticed. In fact, investigations in young competitive swimmers are few in comparison to those carried out in adult/elite swimmers mainly due to financial costs but also to ethical issues. So, we believe that the study of the effects of dry land strength training combined with aerobic training in young competitive swimmers could lead to interesting and useful data in improving overall performance; using a simple combination of swimming specific training and a simple dry land resistance training regimen.

There is strong evidence that school-based interventions are effective in promoting physical activity (PA) levels in youth and therefore school seems to provide an excellent setting to enhance activity levels by implementing physical fitness programs (Sallis et al., 1997; Strong et al., 2005). The school-based Physical Education (PE) interventions can include modified PE classes, generally with more classroom time and more moderate-to-vigorous PA (Mota et al., 2006). In addition, PE classes draw heavily upon muscular strength and endurance (Santos et al., 2011a). On this issue, the scientific literature has produced inconclusive findings (Eliakim et al., 2001). The precise mechanisms that underlie the observed impairments in training adaptation during concurrent training have yet to be identified. Cross-sectional comparisons gave equivocal results relating PA to indicators of muscular strength and endurance (Garrido et al., 2010). Experiments in school environment, concerning this issue, are even scarcer. Further, to our best knowledge, only two studies study had examined the effects of resistance training with concurrent resistance and endurance training on muscular strength development in a sample of adolescent schoolboys (Santos et al., 2011a) and girls (Santos et al., 2011b). So, recently our lab recruited (Santos et al., 2011a) forty two healthy boys from a Portuguese public high school (age: 13.3±1.04 yrs) and divided them into three experimental groups to train twice a week for 8 wk: a resistance training group (GR: n=15), a concurrent resistance and endurance training group (GCOM: n=15) and a control group (GC: n=12; no training program). Significant training-induced differences were observed in 1 kg and 3 kg medicine ball throw gains (GR: +10.3% and +9.8% respectively; GCOM: +14.4% and +7% respectively), whereas no significant changes were observed after a DT period in both the experimental groups. Significant training-induced gains in height and length of the countermovement (vertical and horizontal) jumps were observed in both experimental groups. After training period estimated VO_2_max increased only significantly for GCOM (4,6%, p=0.01). The same authors (Santos et al., 2011b) also compared the effects of an 8-week training period of resistance training alone (GR), or combined resistance and endurance training (GCOM) on body composition, explosive strength and VO_2_max adaptations in a group of adolescent schoolgirls. Sixty-seven healthy girls recruited from a Portuguese public high school (age: 13.5±1.03 years, from 7^th^ and 9^th^ grades) were divided into 3 experimental groups to train twice a week for 8 wk: GR (n=21), GCOM (n=25) and a control group (GC: n=21; no training program). Anthropometric parameters variables as well as performance variables (strength and aerobic fitness) were assessed. No significant training-induced differences were observed in 1 kg and 3 kg medicine ball throw gains (2.7 to 10.8%) between GR and GCOM groups. Therefore, concurrent training seems to be an effective, well-rounded exercise program that can be prescribed as a means to improve muscle strength in healthy schoolboys. Moreover, performing simultaneously resistance and endurance training in the same workout does not impair strength development in young schoolboys and girls, which has important practical relevance for the construction of strength training school-based programs.

## Strength vs. Detraining:

### Elite Team Sports

The maintenance of physical performance during a specific detraining period (decreased in RT volume and/or intensity) may also be explained by the continuation of specific sport practices and competitions and, simultaneously, by the short duration of detraining itself (decreased in RT volume and/or intensity). It is unclear whether the inconsistency of results between different studies involving different sports is due to methodological differences, different training backgrounds, or to different population characteristics. For example, Kraemer et al. (1995) observed that recreationally trained men can maintain jump performance during short periods of detraining (6 weeks). These researchers argued that other factors like jumping technique may be critical for vertical jump performance and may have contributed to the lack of change in jump ability. Marques and González-Badillo (2006) found that professional team handball players declined in jump ability during a detraining period (7 weeks), though not significantly so. This could suggest that game-specific jumping is a better means of positively influencing jump performance. It might be further inferred that game-specific jumping better promotes jump performance amongst those sports where jumping is fundamental. These findings also corroborate our personal professional experience. In fact, reducing ST volume for a short time (2–3 weeks) is not synonymous with performance decline. Occasionally, performance would even increase or at least remain stable.

### Youth populations

Immediately following a 8 weeks a ST program, Garrido et al. (2010) submitted a group age swimmers to 6-week detraining period, maintaining the normal swimming program, without any ST. During this DT period, the subjects performed 33 swimming training units (5.50 ± 0.44 sessions per week). The remaining training comprised low aerobic tasks, technical and velocity training. To the best of our knowledge, this study published by our research team was the first to examine the detraining effects on young swimming athletes (<13 years old). Thus, it is difficult to compare the results with other studies that have investigated strength cessation because they differ markedly in a number of factors, including the sample and the method of measurement. In addition, few studies examined detraining effects in swimming athletes and most of them analyzed physiological parameters variables and not strength or performance variables. On this, Neufer et al. (1987) observed that college swimmers maintained their muscular strength as measured on a swim bench during four weeks of training cessation, but their swim power, i.e., their ability to apply force during swimming, declined by 13.6%. This could be due to a longer period of detraining. It seems that with shorter detraining periods of between 2 to 6–7 weeks, performance could be maintained as was showed on Garrido et al. (2010) who investigated an intervention group. Subjects showed no decline in their swimming performance during the detraining period. As expected, specific swimming training positively influenced sprint swim performance.

More recently, our research team (Santos et al., 2011b) has also studied the effects of a 12-consecutive weeks detrained period during the summer holidays. A sample of healthy boys recruited from a Portuguese public high school were randomly divided into three experimental groups to train twice a week for 8 wk: GR (n=15), GCOM (n=15) and a control group (GC: n=12; no training program). Immediately following this, they commenced a DT period during the summer holidays. Only the GCOMB significantly decreased body weight (−1.7%, p=0.03). There was no significant difference in body mass index on the GR group from post-training to the detraining moment. In addition, there was no significant difference in body fat percentage loss between GR and GCOM during the intervention period.

After post-training moment, all groups showed no significant loss performance on jump performance (Table 2). In speed running a significant loss performance was expected but was not found in both GR and GCOM. In the 1 and 3 kg medicine ball throw distance test, no significant changes were observed for experimental an group, which signifies a sustained effect of training in this explosive task. Our results are in disagreement with the findings of Ingle et al. (2006) where over a detraining 12 week period the experimental group saw significant reductions for all of the resistance exercises that ranged from 16.3 to −30.3%. Control group had also no differences in performance marks for both 1 and 3kg medicine ball throw distance test. Therefore, it must be suggested that explosive strength gains induced by both training programs were kept after a DT period of 12 weeks, as strength is determined, among other factors, by muscular mass.

Faigenbaum et al. (1996) showed that 8 weeks of detraining led to significant losses of leg extension (−28.1 %) and chest press (−19.3%) strength whereas control group strength scores remained relatively unremarkable. Finally, the VO_2_max (ml.kg^−1^.min^−1^) remained stable for GCOM, except for GR where a significant loss (−6.8%) was observed. Another study (Santos et al., 2011a) found that changes are more moderate in recently trained subjects (compared with highly trained subjects) in the short-term, but recently acquired VO_2_max gains are completely lost after training stoppage periods longer than 4 weeks. Conversely, our results show that GCOM kept VO_2_max gains even after 12 weeks of DT. The detraining effect over VO_2_max has been poorly studied in non-adult and non-sporting samples.

In summary, the latest studies have demonstrated that high level athletes from different sports can enhance strength values using moderate overall volume.

However, it is our opinion that further studies are still necessary to clarify this statement. Furthermore, our results suggest that a concurrent resistance and endurance training program seems markedly effective on both strength and endurance fitness features of school-age boys and girls. Thus, simultaneously performing resistance and endurance training in the same workout does not impair strength development in age-group athletes, which has important practical relevance for the construction of ST programs.

## Figures and Tables

**Table 1:** Training Efficiency

	AI per-exercise (%)		Total reps		ATE (% lift^−1^)
Exercises	1^st^ cycle	2^s^ cycle	1^st^ cycle	2^s^ cycle	1^st^ cycle & 2^s^ cycle
					
1RMBP	82.7	82.5	124	87	**0.14**
4RMPS	85.9	86.2	159	111	**0.16**

*(after Marques and González-Badillo, 2006). AI: average intensity Total reps: The total number of repetitions (sets x reps) lifted at loads greater than 80% of 1RMBP and 4RMPS, respectively. ATE (average training efficiency): The average percentage gain in bench press and squat performances during the 12 training period divided by the total number of repetitions lifted at loads greater than 80% of 1RMBP and 4RMPS, respectively. 1RMBP: concentric 1- repetition maximum bench press; 4RMPS: 4- repetition maximum parallel squats 1^st^ cycle: 1*–*6 weeks; 2^s^ cycle: 7*–*12 weeks.*

**Table 2:** Mean ± standard deviation of upper and lower limbs strength values at all testing trials

		M1	M2	

	Group	x±σ	x±σ	p value (M1–M2)
CM Vertical Jump (cm)	GC	0,317±0,07	0,317±0,09	0,71
	GR	0,306±0,07	0,277±0,08	0,14
	GCOM	0,316±0,09	0,295±0,10	0,37
CM Horizontal Jump (cm)	GC	1,63±0,33	1,62±0,51	0,72
	GR	1,56±0,30	1,47±0,36	0,17
	GCOM	1,74±0,32	1,54±0,43	0,12
1kg Medicine ball throwing (m)	GC	8,31±1,71	8,89±1,75	0,11
	GR	8,15±1,62	8,13±1,45	0,31
	GCOM	7,59±1,73	7,71±2,27	0,37
3kg Medicine ball throwing (m)	GC	5,01±1,19	5,35±1,30	0,15
	GR	5,12±1,08	5,10±0,99	0,29
	GCOM	5,11±1,17	5,03±1,25	0,97
Running Speed 20m (s)	GC	4,12±0,48	3,52±0,49	0,12
	GR	4,05±0,42₮	4,04±0,36	0,43
	GCOM	3,81±0,28	3,83±0,50	0,93
VO_2_Max (mL.kg^−1^.min^−1^)	GC	47,4±5,5	44,4±8,1	0,52
	GR	46,8±6,5	42,1±5,2	0,04
	GCOM	51,2±6,7	51,7±6,6	0,83

(after Santos et al., 2011a, in press)
